# Editorial: Neuropsychiatric disorders in the veterans volume II: emerging evidence of precision medicine and complementary and integrative health (CIH) approaches

**DOI:** 10.3389/fpsyt.2024.1456022

**Published:** 2024-07-18

**Authors:** Giulio M. Pasinetti, J. Wesson Ashford, Andrea Durham, Divyash Shah, Vrinda Saxena, Joshua Palmieri

**Affiliations:** ^1^ Department of Neurology, Mount Sinai School of Medicine, New York, NY, United States; ^2^ Geriatric Research, Education and Clinical Center, James J. Peters Veterans Affairs Medical Center, Bronx, NY, United States; ^3^ War Related Illness and Injury Study Center, Veterans Affairs (VA) Palo Alto Health Care System, Palo Alto, CA, United States; ^4^ Department of Psychiatry & Behavioral Sciences, Stanford University, Palo Alto, CA, United States

**Keywords:** veterans, neuropsychiatric disorders, mental health barriers, precision medicine, complementary and integrative health, PTSD

## 1 Introduction

Veterans protect and serve their country, but alongside these responsibilities lies an unfortunate reality that many suffer from neuropsychiatric disorders that are not fully addressed. The nature of their work often involves exposure to potentially traumatic experiences, like violence and warfare, especially while in combat. Among other factors, the work and training that veterans were previously subject to requires responsibility, sacrifice and unrelenting commitment, and subsequently servicemen and women may be pushed to physical and mental extremes in the process. Consequently, following exposure to potentially morally injurious events (PMIEs), moral injury can manifest itself in the form of psychological, behavioral, and social distress (1). Their experiences can linger with them even after returning from their duties and can occur concommitantly with psychological and emotional burdens. Thus, it is critical to consider ways to assess veterans and treat their unique psychiatric disorders. This Research Topic presents novel approaches and study designs to identify, assess, and mitigate a variety of neuropsychiatric disorders, which are of particular concern for the veteran population.

## 2 Addressing mental health in the veteran population

Understanding and assessing the mental health of our servicemen and women and how it is influenced by their work is crucial. Suicide is a serious issue and high rates are of growing concern among the veteran population (2). Thousands of veterans lose their lives each year to suicide (2). Although veterans only comprise 7.6% of the US population, they are disproportionately affected by suicide, accounting for almost 14% of American adult suicides (3).Veterans are exposed to trauma, isolation, stress, and accessibility to firearms which are noted as some of the possible explanations for veterans being disproportionately affected by suicide (4). Along with suicide, depression is regarded as a common issue among military veterans and may even influence suicidal behavior in conjunction with post-traumatic stress disorder (PTSD) (5). Higher rates of depression are observed in the veteran population, which is usually linked to the experiences that they faced during combat (6).

PTSD and substance use disorders (SUDs) are other mental health issues that can take a significant toll on the daily lives of veterans, yet many do not actively engage in treatment strategies (7). PTSD is a disorder associated with impairment in emotional and cognitive responses in response to traumatic events, which can manifest in forms of avoidance, fear, and unwanted memories or emotions (8). PTSD is often a prevalent concern among veterans especially because of experiences relating to the brutality of war during military service (8). As for SUDs, opioid overdose among veterans remains a substantial problem. It has been noted that between 2010-2019, drug overdose mortality rates increased by over 50% among US veterans (9). Opioid dependence among veterans may be facilitated by combat related injuries and the liberal prescription of these substances, though there are amalgamations of additional biological, psychological, and social factors that can explain the risk of opioid use disorder (OUD) among military personnel (9). All of these mental health issues result in a contextualized, intricate combination that is specific to military personnel, in part, due to their unparalleled experiences in combat.

## 3 Strategies to mitigate neuropsychiatric disorders in veterans

The articles in this Research Topic capture diverse perspectives and propose novel approaches and strategies that can be applied to understand, address, and improve veteran mental health struggles. These studies present methods that emphasize personalized health, precision medicine, and complementary and integrative health (CIH) to improve a veteran’s well-being. For instance, Doenyas-Barak et al. identify hyperbaric oxygen therapy (HBOT) as a potential therapeutic intervention for veterans with PTSD by drawing on clinical data and discuss the biological mechanisms of HBOT that may explain the efficacy of this treatment strategy. Three features in this topic highlight randomized controlled trial approaches to assess treatment strategies relating to psychological health in veterans. Peterson et al. provide a study protocol that highlights the methodology and design of a flexibility training intervention approach to address and prevent psychiatric symptomatology in veterans by promoting resilience and readiness prior to onset of symptoms. Mathersul et al. apply a personalized medicine approach through a randomized-clinical trial design to assess heart rate variability (HRV) and self-reported emotion regulation as non-specific predictors and treatment moderators, as it relates to PTSD treatment in veterans and ultimately propos HRV to be a useful biomarker to assess and optimize PTSD treatment strategies. Mumba et al. present results from another randomized control trial that shows how a greater adherence to medications for opioid use disorder (MOUD) can be achieved through a combination of peer support and mindfulness-based relapse prevention, which has implications for addressing the growing concern of OUD in the veteran population. Kindt and Soeter propose a personalized treatment approach for veterans with PTSD that incorporates context related to their trauma to make therapeutic interventions more successful for patients using an open-label case-series design. Though these studies have different methodologies, they all underscore the need for new approaches that can be applied to better treat the psychiatric disorders currently affecting veterans.

## 4 Conclusion

Accounting for the mental health of our servicemen and servicewomen is a critical area of concern that must not be underestimated. The unique experiences of military personnel, whether in combat or training, may make them more vulnerable to psychological distresses that are not necessarily common among civilian life. Veterans make significant contributions to their countries, and along with their duties to serve comes a cost related to psychological and mental health burdens that must be acknowledged and managed. The features in this Research Topic illuminate several neuropsychiatric disorders and propose novel therapeutic CIH interventions that can be applied to support and advance our treatments of neuropsychiatric health in veterans.

## Author contributions

GP: Conceptualization, Project administration, Supervision, Writing – original draft, Writing – review & editing. JA: Conceptualization, Project administration, Supervision, Writing – original draft, Writing – review & editing. AD: Conceptualization, Writing – original draft, Writing – review & editing. DS: Conceptualization, Writing – original draft, Writing – review & editing. VS: Conceptualization, Project administration, Writing – original draft. JP: Conceptualization, Project administration, Writing – original draft.
